# Seismic Response Mitigation of a Television Transmission Tower by Shape Memory Alloy Dampers

**DOI:** 10.3390/ma14226987

**Published:** 2021-11-18

**Authors:** Jingbo Wu, Bo Chen, Lunhai Zhi, Xinxin Song

**Affiliations:** 1 Key Laboratory of Roadway Bridge and Structural Engineering, Wuhan University of Technology, Wuhan 430070, China; wujingbo0618@whut.edu.cn (J.W.); songxinxinfx@163.com (X.S.); 2 School of Civil Engineering, Hefei University of Technology, Hefei 230009, China; zhilunhai1979@163.com

**Keywords:** television transmission tower, seismic excitation, shape memory alloy damper, parametric study, vibration control

## Abstract

High-rise television transmission towers are of low damping and may vibrate excessively when subjected to strong earthquakes. Various dynamic absorbers and dampers are proposed to protect television transmission towers from excessive vibrations and damages. Up to now, the seismic damage reduction in television towers, using SMA dampers under seismic excitations, has not been conducted. To this end, the response reduction in a flexible television tower, disturbed by earthquakes using SMA dampers, is conducted in this study. A two-dimensional dynamic model is developed for dynamic computation at first. The mathematical model of an SMA damper is proposed, and the equations of motion of the tower, without and with, are established, respectively. The structural dynamic responses are examined in the time and the frequency domain, respectively. The effects of damper stiffness, service temperature, hysteresis loops, and earthquake intensity on control efficacy are investigated in detail. In addition, the power spectrum density curves, of dynamic responses and the energy responses, are compared to provide deep insights into the developed control approach. The control performance of SMA dampers is compared with that of widely-used friction dampers. The analytical observations indicate that SMA dampers with optimal parameters can substantially reduce the vibrations of TV transmission towers under seismic excitations.

## 1. Introduction

To be important high-rise infrastructures, television (TV) transmission towers are widely used across the world for TV transmission and sightseeing. These TV transmission towers, with low damping, may vibrate excessively in severe environments, inducing possible damage and even failure [1,2]. For instance, a real steel TV transmission tower, with a height of 136m, in China collapsed under strong winds in 2012 [3]. The collapse of transmission towers subjected to strong earthquakes in China is also reported [4]. The simplest approach to reduce vibration of TV transmission towers is to increase inherent structural ductility, although it is often uneconomical. Thus, various control devices, such as dynamic absorbers and energy-dissipating dampers, are developed to protect TV transmission towers from excessive vibrations and damages [5,6,7]. The dynamic absorbers are firstly applied in the vibration control of high-rise towers. The wind-induced responses of high-rise towers are studied by Hitchcock et al. [8] and Balendra et al. [9], using tuned liquid dampers and active tuned liquid column dampers. Similar studies are carried out using tuned mass dampers (TMD), such as Yang et al. [10] for a steel tower, Wu and Yang [11] for the Nanjing TV tower, Ghorbani-Tanha et al. [12] for the Milad Tower, and Lu et al. [13] for Shanghai World Financial Center Tower. An obvious defect of dynamic absorbers for vibration control of TV transmission towers is the large space requirement, which often conflicts with the structural function in operation.

As an alternative approach, friction dampers and fluid dampers have been utilized in the structural vibration control of truss towers for many years [14,15,16,17]. Chen et al. [18] conducted the seismic response mitigation of TV transmission towers by using friction dampers. Similar control approaches have been developed by fluid dampers due to their simple configuration and low cost. For instance, Zhang and Li [19] investigated the responses, of a flexible tower under earthquakes, by fluid viscous dampers. Recently, smart materials are widely used in the field of vibration engineering to fabricate various smart dampers for structural vibration control. Xu et al. [20] carried out the semi-active control of a wind-excited tower by piezoelectric friction dampers, associated with a local feedback control algorithm. Chen et al. [21] conducted the semi-active control of a flexible tower subjected to winds using magnetorheological dampers.

A typical smart material, shape memory alloy (SMA) has been recently gaining great popularity due to the properties of high strength, super-elasticity, energy dissipation, better fatigue, and corrosion resistance [22,23,24,25,26]. SMA can exhibit excellent super-elasticity, and this feature is explored to fabricate SMA energy dissipation systems, including SMA bracings, SMA dampers, and connection elements, for vibration control. Zheng et al. [27] proposed a self-adaptive Ni-Ti SMA-cable-based frictional sliding bearing to mitigate pounding and unseating damage of bridges subjected to earthquakes. Liu et al. [28] investigated the seismic response control of a steel frame using base isolation systems with SMA springs.

However, the seismic response suppression, of TV transmission towers with SMA dampers, has not yet been studied. In this regard, the seismic behavior of TV transmission towers with SMA dampers is examined in this study. The two-dimensional (2D) lumped mass model is developed based on the three-dimensional (3D) finite element (FE) model for structural dynamic computation. The model of an SMA damper is proposed, and the equations of motion of the tower, without and with control, are established, respectively. Three schemes are studied and compared to determine the rational location of SMA dampers. The effects of damper stiffness, service temperature, hysteresis loops, and earthquake intensity on control efficacy are examined through a detailed parametric study. In addition, the power spectrum density (PSD) curves of dynamic responses are examined to explore the vibration properties in the frequency domain. The control performance of SMA dampers is compared with that of widely-used friction dampers. The energy responses of the uncontrolled and controlled towers are also compared to provide deep insights into the developed control approach. The made observations indicate that SMA dampers with optimal parameters are beneficial to the vibration control of TV transmission towers under seismic excitations.

## 2. Seismic Responses of a TV Transmission Tower

**Figure 1:**
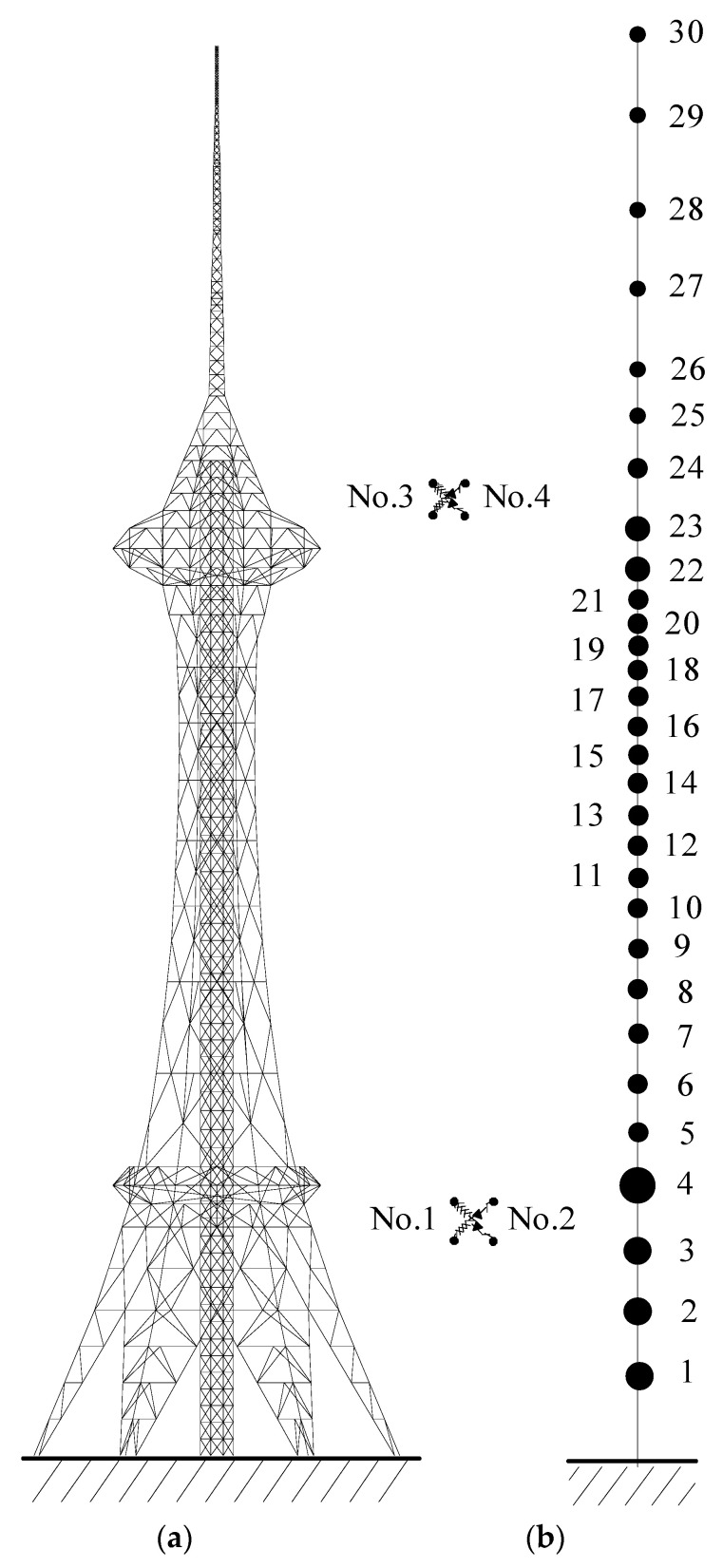
Analytical model of a TV transmission tower. (**a**) 3D FE model; (**b**) 2D dynamic model.

The 3D FE model of the TV transmission tower is firstly constructed with the aid of the commercial package ABAQUS, as displayed in Figure 1a. The configuration of the TV transmission tower is quite complicated, and the number of the dynamic degree-of-freedoms (DOFs) of the 3D FE model is quite large. Thus, the dynamic response computation is time-consuming, and the displacement-force relationship of SMA dampers is nonlinear. The dynamic analysis of the tower-damper system needs a lot of iterations at each time step. Thus, the response computation and parametric study on control performance are quite tedious and time-consuming for a complicated 3D FE model [18,20]. To this end, 2D lump models, with satisfactory accuracy, are commonly utilized to simplify dynamic computation and parametric study [18,20], as shown in Figure 1b. Thirty nodal floors are determined, and each floor is assumed to have only one dynamic DOF. The stiffness matrix is determined based on the flexibility matrix of the original 3D FE model.

During the construction of the 2D lumped dynamic model of the TV transmission tower, the structural masses are concentrated at limited nodal floors, and the mass matrix of the reduced 2D dynamic model is established using a lumped mass matrix. The stiffness matrix of the 2D lumped model can be determined based on the flexibility matrix of the 3D FE model of the tower. Apply the same horizontal unit force at the thirty node floors one by one and compute the flexibility coefficient of each floor. Then, form the flexibility matrix of the tower and inverse the flexibility matrix to obtain the stiffness matrix of the 2D model.

## 3. Model of SMA Damper

The model of SMA material can be described based on thermodynamic theory, and the commonly accepted constitutive model is:

(1) $$\sigma -\sigma_{0}=E\left(\xi \right)\varepsilon -E\left(\xi_{0}\right)\varepsilon_{0}+\Omega \left(\xi \right)\xi_{S}-\Omega \left(\xi_{0}\right)\xi_{S0}+\Theta \left(T-T_{0}\right)$$

where *σ* and *ε* are the stress and strain of the SMA wire, respectively; *σ*_0_ and *ε*_0_ are the stress and strain of the SMA wire at the initial state, respectively; *ξ* is the volume percentage of the martensite; *ξ*_0_ is the martensite content of the SMA wire at initial state; *T*_0_ is the temperature of the SMA wire at initial state; *ξ_S_*_0_ is the initial volume content of the martensite; *ξ_S_* is the volume content of the martensite; *E* is the Young’s modulus of SMA material; Ω is the phase transition tensor; Θ is the thermo-elastic tensor of SMA material; *T* is the temperature at a reference state. 

The Young’s modulus of an SMA wire, related to the martensite content *E*(*ξ*), is given by

(2) $$E\left(\xi \right)=\xi E_{M}+\left(1-\xi \right)E_{A}$$

where *E_A_* and *E_M_* are Young’s moduli at the austenite and martensite phases, respectively. 

**Figure 2:**
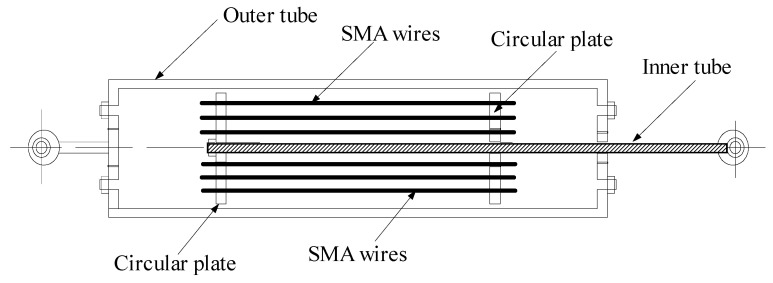
Schematic configuration of SMA damper.

The inherited properties of SMA wires, such as self-centering, better fatigue, and corrosion resistance, are useful in the design of energy-dissipating dampers. An SMA damper with re-centering capacity is designed according to the super-elasticity and high damping characteristics of the SMA wire, as shown in Figure 2. The SMA damper consists of the outer tube, inner tube, SMA wires, and two circular plates. The SMA wire is designed always in tension when the damper works. The SMA wire can dissipate vibrant energy, during its reciprocal movement, to suppress the structural vibration.

**Figure 3:**
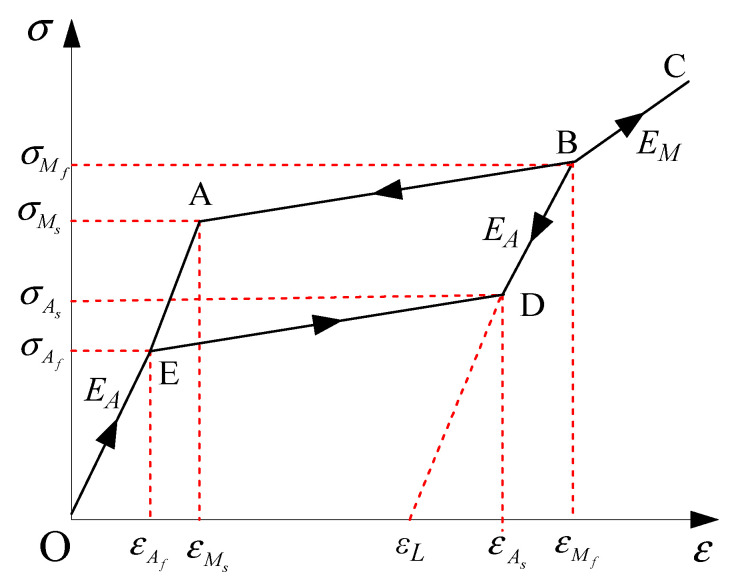
Flag-shaped hysteretic model of an SMA wire.

The hysteretic model of the Ni-Ti SMA material is displayed in Figure 3. In the figure, *M_f_* and *M_s_* denote the martensite finish temperature and martensite starts temperature, respectively. *A_f_* and *A_s_* denote the austenite finish temperature and austenite start temperature, respectively; $\sigma_{M_{s}}$ and $\varepsilon_{M_{s}}$ represent the critical stress and strain of the Ni-Ti SMA material at *M_s_*, respectively; $\sigma_{M_{f}}$ and $\varepsilon_{M_{f}}$ are the critical stress and strain at *M_f_*, respectively; $\sigma_{A_{s}}$ and $\varepsilon_{A_{s}}$ are the critical stress and strain at *A_s_*, respectively; $\sigma_{A_{f}}$ and $\varepsilon_{A_{f}}$ are the critical stress and strain at *A_f_*, respectively; *ε_L_* is the maximum residual strain. 

When the working temperature of the SMA material is given, the mechanical property of the SMA material will be known [29,30,31]. As illustrated in Figure 3, the relationships of strain and stress, on different paths associated with Ni-Ti SMA material, can be expressed as follows:

(1) Paths O-A and E-O are elastic (full austenite):
  (3) $$\sigma (t)=E_{A}\varepsilon (t)$$
  The control force of the SMA damper *u*(*t*) is:
  (4) $$u(t)=\frac{E_{A}A}{l_{s}}(x_{}^{d}(t)-l_{s})$$
  where $x_{}^{d}(t)$ is the length of the damper after deformation; *A* is the cross-sectional area of SMA wires; *l_s_* is the initial length of the SMA wires.
(2) Path A-B (forward transformation):
  (5) $$\sigma (t)=\sigma_{M_{s}}+\frac{\sigma_{M_{f}}-\sigma_{M_{s}}}{\varepsilon_{M_{f}}-\varepsilon_{M_{s}}}(\varepsilon (t)-\varepsilon_{M_{s}})$$
  (6) u(t)=σ(t)A
(3) Path B-C is elastic (full martensite):
  (7) $$\sigma (t)=\sigma_{M_{f}}+E_{M}(\varepsilon (t)-\varepsilon_{M_{f}})$$
  (8) $$u(t)=\frac{E_{M}A}{l_{s}}(x^{d}(t)-l_{s})$$
(4) Path B-D is elastic (full martensite)
  (9) $$\sigma (t)=\sigma_{M_{f}}+E_{M}(\varepsilon (t)-\varepsilon_{M_{f}})$$
  (10) $$u(t)=\sigma_{M_{f}}A+E_{M}\left[(x_{}^{d}(t)-l_{s})-\varepsilon_{M_{f}}\right]A$$
(5) Path D-E (reverse transformation):
  (11) $$\sigma (t)=\sigma_{M_{s}}+\frac{\sigma_{M_{s}}-\sigma_{A_{f}}}{\varepsilon_{A_{s}}-\varepsilon_{A_{f}}}(\varepsilon (t)-\varepsilon_{A_{s}})$$
  (12) u(t)=σ(t)A
  in which *σ*(*t*) and *ε*(*t*) are the stress and the strain of SMA wires, respectively.

## 4. Dynamic Analysis of the Controlled Tower

The equations of motion of the controlled tower with SMA dampers is given by:

(13) $$M\ddot{x}(t)+C\dot{x}(t)+Kx(t)=-MI_{g}{\ddot{x}}_{g}(t)+Hu(t)$$

(14) $$u(t)=[\begin{array}{cccc} u_{1} & u_{2} & \cdots & u_{m} \end{array}]$$

where x(t), $\dot{x}(t)$, and $\ddot{x}(t)$ are the displacement, velocity, and acceleration responses of the TV transmission tower, respectively; **M**, and **K** are the mass and stiffness matrices of the tower, respectively; **C** is the Rayleigh damping matrix; **I***_g_* is the influence vector for ground acceleration; ${\ddot{x}}_{g}(t)$ is the seismic acceleration; **H** is the position matrix of control force; **u**(*t*) is the control force of SMA dampers; *m* is the number of SMA dampers.

The incremental equations of the TV transmission tower are given by:

(15) $$M\Delta \ddot{x}(t)+C\Delta \dot{x}(t)+K\Delta x(t)=-MI_{g}\Delta {\ddot{x}}_{g}(t)+H\Delta u(t)$$

where Δx(t), $\Delta \dot{x}(t)$, and $\Delta \ddot{x}(t)$ are the displacement increment, velocity increment, and acceleration increment, respectively; $\Delta {\ddot{x}}_{g}(t)$ is the increment of the ground acceleration.

The above incremental equation is solved by the Newmark-*β* method with a time step of Δ*t*. It is noted that the increment vector of SMA dampers Δ**u**(*t*) can only be determined under the known Δ**x**(*t*) and $\Delta \dot{x}(t)$ in the numerical computation. However, the displacement increment Δ**x**(*t*) and velocity increment $\Delta \dot{x}(t)$ is commonly determined based on Δ**u**(*t*). Thus, a dynamic iteration process is developed to ensure computational convergence. The iteration process proceeds until Δ**x**(*t*) satisfies the following equation:

(16) $$abs[(\Delta x{\left(t\right)}^{\left(i+1\right)}-\Delta x{\left(t\right)}^{\left(i\right)})/\Delta x{\left(t\right)}^{\left(i\right)}]<\kappa$$

where κ is a prescribed error limitation related to an actual TV transmission tower. 

Energy response is an important index to describe the vibration of TV transmission towers. The energy representation of the example tower is formed by integrating Equation (15). The absolute energy equation of the uncontrolled tower is given by:

(17) $$E_{KE}+E_{DE}+E_{SE}=E_{E}$$

Similarly, the energy equation for the tower with SMA dampers is:

(18) $$E_{KE}+E_{DE}+E_{SE}+E_{PE}=E_{E}$$

where *E_E_* is the total input energy from earthquakes; *E_KE_* and *E_SE_* are kinetic energy and strain energy; *E_DE_* and *E_PE_* are the energy dissipated by structural damping and SMA dampers, respectively.

## 5. Case Study

### 5.1. Analytical Parameters

**Figure 4:**
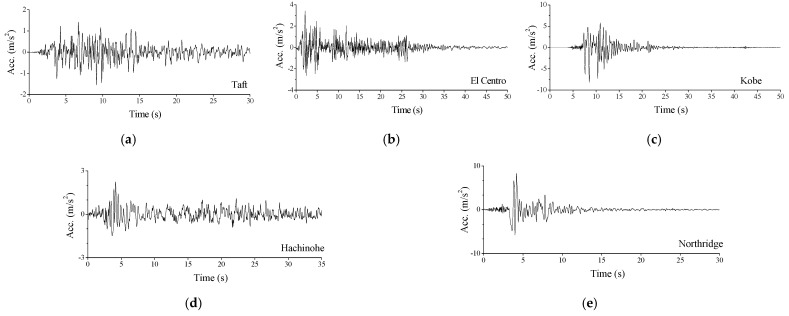
Time histories of four historical seismic records. (**a**) Taft; (**b**) El Centro; (**c**) Kobe; (**d**) Hachinohe; (**e**) Northridge.

A real TV tower, with a height of 340 m, is used to examine the feasibility of the proposed control method, as displayed in Figure 1. Two turrets are developed for sightseeing. The 3D model of the TV tower is established by ABAQUS, and then, a 2D dynamic model is constructed by a developed computer program. The validity of the 2D model is examined by comparing structural dynamic characteristics, as listed in Table 1. The fundamental frequency of the 3D and 2D models are 0.129 Hz and 0.128 Hz, respectively, with an error of 0.621%. It is also found that the average error of the first eight natural frequencies is 3.641%. Thus, the dynamic characteristics of the 2D dynamic model are close to that of the original 3D model, and the validity of the 2D model can be guaranteed. To evaluate the effectiveness of the proposed control method, three far-field and two near-field historical records are selected [32,33,34,35]: (1) Taft earthquake on 21 July 1952; (2) El Centro earthquake on 18 May 1940; (3) Kobe earthquake on 17 January 1995; (4) Hachinohe earthquake on 16 May 1968; (5) Northridge earthquake on 17 January 1994, respectively. The original peak ground accelerations (PGAs) of the five seismic records are 1.559, 3.417, 8.178, 2.250, and 8.2676 m/s^2^, as shown in Figure 4. All the earthquakes are scaled to have the same PGA of 4.0 m/s^2^ for comparison. The time step and damping ratios in the dynamic analysis are 0.02 s and 0.01, respectively. 

Obvious whiplash effects of the mast are observed based on analytical results. Owing to the small geometric size of the mast, sixty SMA dampers are evenly divided into four groups and installed around the two turrets. Thirty SMA dampers (group nos. 1 and 2) are installed at the low turret, and the other two groups of SMA dampers are at the upper turret. An SMA damper with an axial brace connects spherical nodes in the axial direction of a member. The physical parameters of SMA dampers are listed in Table 2. The Young’s modulus of the SMA damper brace is 2.12 × 10^8^ kN/m, and the cross-sectional area of the SMA damper is 50 cm^2^.

### 5.2. Control Performance Comparison

The vibration reduction factor (VRF) is used in this study to evaluate the control efficacy of SMA dampers:

(19) $$\mathrm{VRF}=\frac{R_{nc}-R_{co}}{R_{nc}}$$

where *R_nc_* and *R_co_* are the peak response without and with SMA dampers, respectively.

**Figure 5:**
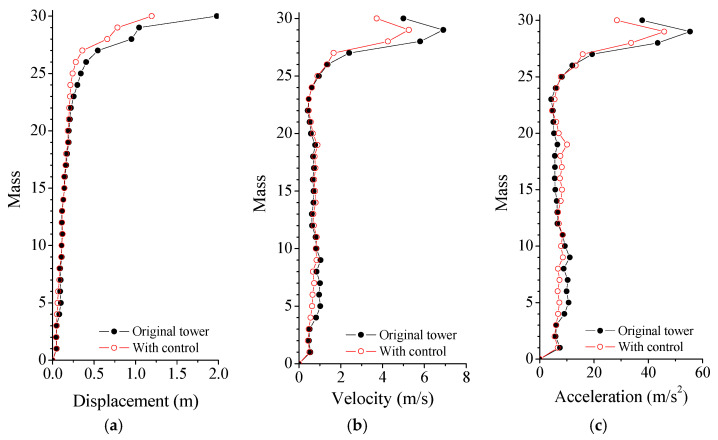
Maximum responses of the tower without/with control. (**a**) Displacement; (**b**) Velocity; (**c**) Acceleration.

**Figure 6:**
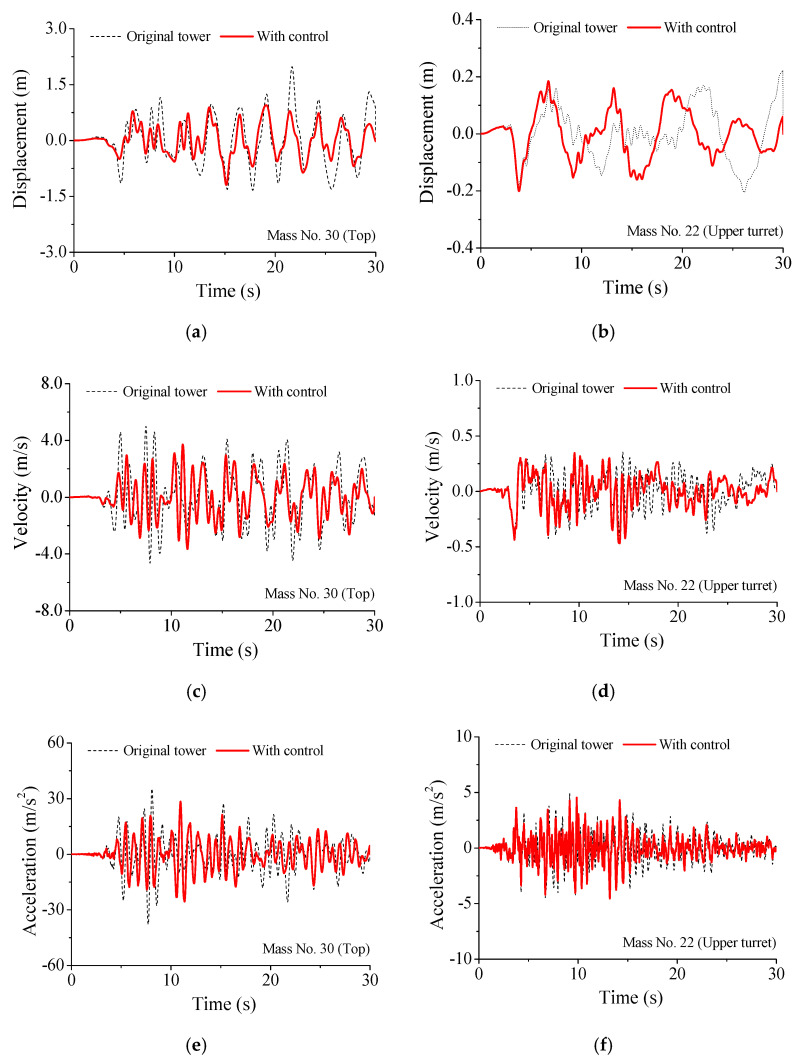
Time histories of dynamic responses of the television tower. (**a**) Displacement No. 30; (**b**) Displacement No. 22; (**c**) Velocity No. 30; (**d**) Velocity No. 22; (**e**) Acceleration No. 30; (**f**) Acceleration No. 22.

The curves in Figure 5 indicate that the peak responses are remarkably mitigated using SMA dampers. It is found that the displacement VRFs of the tower top, upper turret, and low turret are 42.739%, 9.420%, and 37.856%, respectively. Similarly, the counterparts of the acceleration are 28.937%, 9.533%, and 26.405%, respectively. In addition, the displacement and acceleration VRFs are slightly better than those of velocity and responses. As a result, satisfactory control can be achieved using SMA dampers, particularly for the whiplash effects of the mast. The dynamic responses of the tower top and the upper turret, with and without SMA dampers under the Taft earthquake, are displayed in Figure 6. Owing to the additional damping produced by SMA dampers, the vibrations of the controlled tower damped out more quickly than that of the original tower. The application of SMA dampers can mitigate the seismic responses of the TV transmission tower to a great extent. In particular, the control efficacy on whiplash effects of the mast is better than that of the other parts of the tower. 

Three damper installation schemes are considered to compare the effects of damper position on control efficacy, as listed in Table 3. Scheme 1 is mentioned above, in which sixty dampers are around the two turrets. For scheme 2, all the SMA dampers are installed around the low turret (Mass No. 4). For scheme 3, all the SMA are installed around the upper turret. 

**Figure 7:**
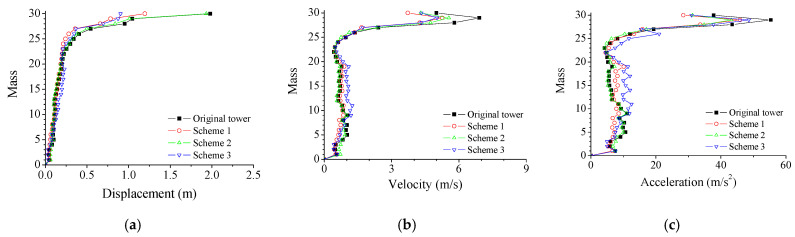
Comparison of different control schemes. (**a**) Displacement; (**b**) Velocity; (**c**) Acceleration.

Figure 7 shows the structural peak responses of different control schemes. For scheme 2, with all the dampers around the low turret, the control efficacy of displacement responses is much better than that of velocity and acceleration responses. Similar observations can be made for scheme 3, whose control performance is slightly inferior to that of scheme 2. The overall control efficacy of scheme 1 is better than that of schemes 2 and 3. Thus, the optimal positions for SMA dampers are both two turrets instead of a single turret. It is also observed that the control efficacy of displacement of all the schemes is slightly better than that of velocity and acceleration responses. Therefore, scheme 1 is adopted for the parametric study in the following sections.

**Figure 8:**
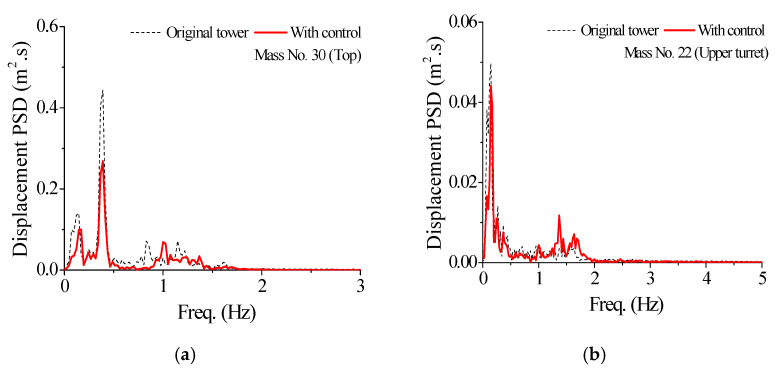

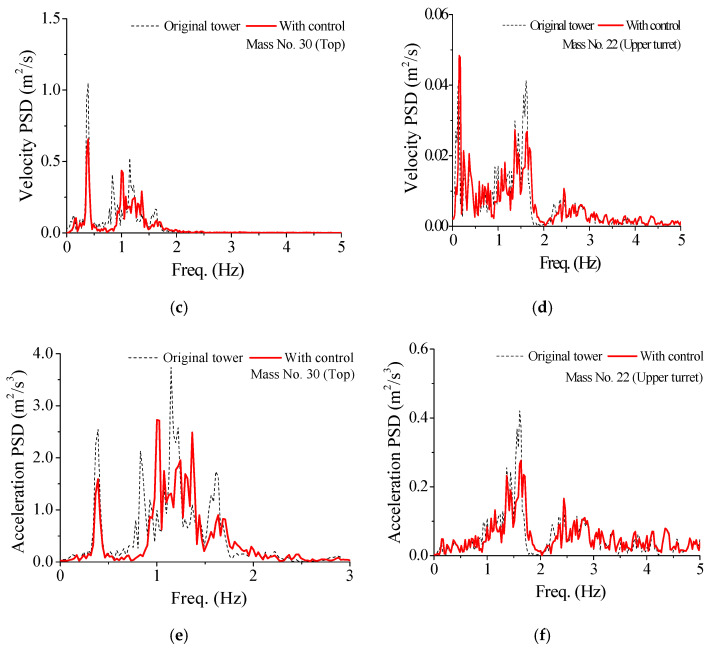
Comparison of PSD curves of response time histories. (**a**) Displacement PSD No. 30; (**b**) Displacement PSD No. 22; (**c**) Velocity PSD No. 30; (**d**) Velocity PSD No. 22; (**e**) Acceleration PSD No. 30; (**f**) Acceleration PSD No. 22.

The control efficacy of SMA dampers under the Taft earthquake is also analyzed and demonstrated in the frequency domain. The power spectral density (PSD) curves of dynamic responses of the tower top and upper turret are displayed in Figure 8. The magnitudes of the PSD curves, for all the three types of responses with control, are much smaller compared with those without control. In particular, the PSD VRFs of the first vibration mode are larger than those of other higher modes. 

## 6. Parametric Study on Control Performance

### 6.1. Stiffness Effects of SMA Damper

The stiffness coefficient of an SMA damper is given by:

(20) $$\mathrm{SC}=\frac{k_{d}^{SMA}}{k_{0}^{SMA}}$$

where $k_{d}^{SMA}$ is the stiffness value in computation; $k_{0}^{SMA}$ is the initial stiffness of an SMA damper. 

**Figure 9:**
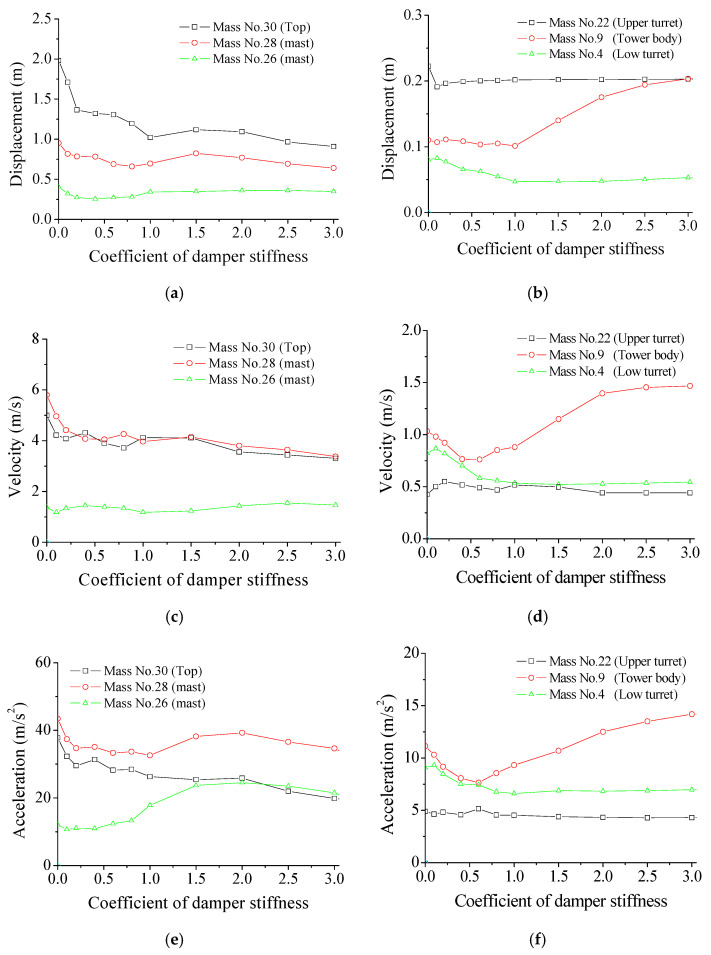
Effects of damper stiffness on peak responses. (**a**) Displacement at mast; (**b**) Displacement at tower; (**c**) Velocity at mast; (**d**) Velocity at tower; (**e**) Acceleration at mast; (**f**) Acceleration at tower.

The stiffness effects of SMA dampers on the peak responses under the Taft earthquake are investigated, and the results are displayed in Figure 9. The peak displacement and velocity responses of the mast are gradually reduced with an increasing SC until approximately 1.0. A further increase in stiffness of SMA dampers does not generate further significant response reduction when SC is larger than 1.0. The peak acceleration of the mast reduces at first and then increases with the increasing SC, which means there exist optimal SC values for structural peak responses. It is not beneficial to set a very large SC value to avoid unnecessary cost waste. Similar results can be achieved from the dynamic responses of the tower body and turrets. Thus, an optimal SC value for all the responses does not exist, and optimal SC values for three kinds of peak responses, at different places, are different to some extent. The optimum SC value for the example tower is selected as 1.0 considering the overall control efficacy of the tower. 

### 6.2. Properties of Hysteresis Loops

**Figure 10:**
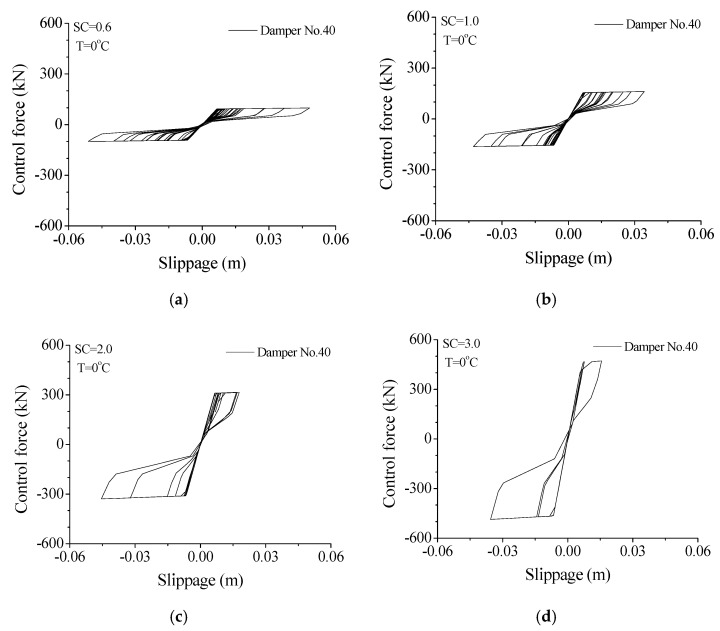
Variation of hysteresis loop with damper stiffness. (**a**) SC = 0.6; (**b**) SC = 1.0; (**c**) SC = 2.0; (**d**) SC = 3.0.

The hysteresis loop is an important index to describe the energy-dissipating capacity of an SMA damper under earthquakes. The variation of a hysteresis loop with damper stiffness is displayed in Figure 10. A small SC value (SC = 0.6) reflects the small enclosed area of the hysteresis loop. Thus, the SMA damper is easy to extend while the energy-dissipating capacity is poor. Increasing the damper stiffness (SC = 1.0 or 2.0) can improve the shape of the hysteresis loops and the energy-absorbing capacity. After the SC value reaches 2.0, a further increase (SC = 3.0) in damper stiffness may increase the peak control force but reduce the enclosed area of the hysteresis loop. Therefore, an optimal SC value of an SMA damper can also be determined based on the configuration of hysteresis loops. 

### 6.3. Effect of Service Temperature

**Figure 11:**
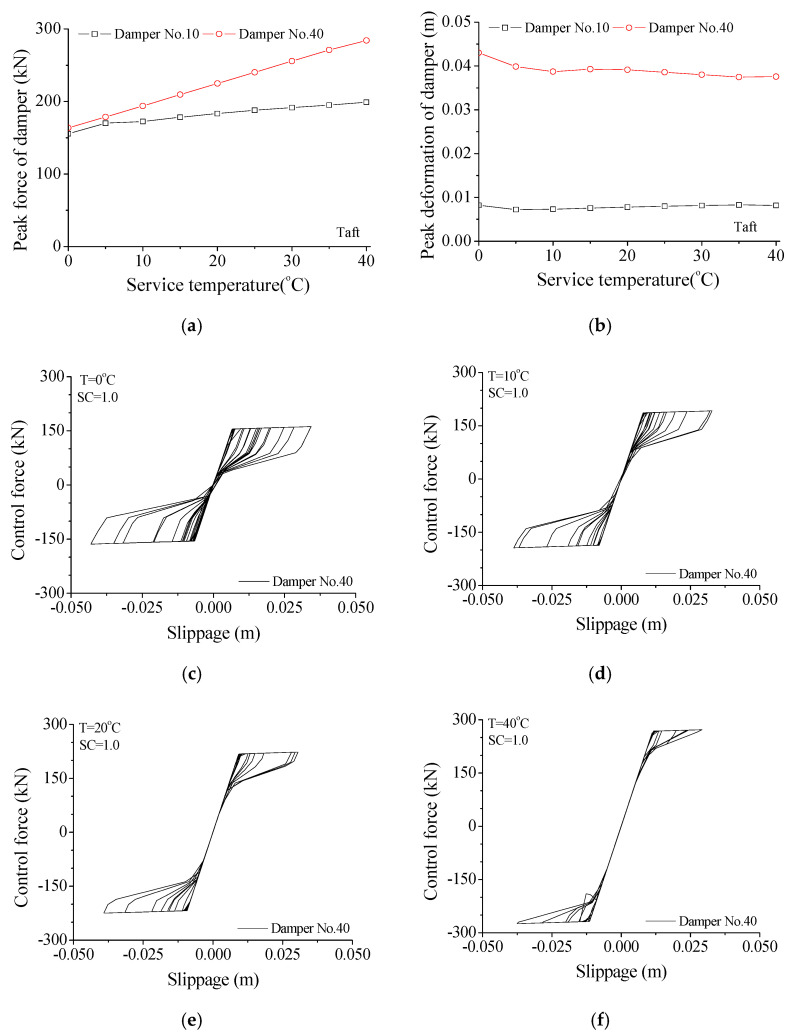
Variation of damper force and deformation with service temperature. (**a**) Peak force; (**b**) Peak deformation; (**c**) T = 0 °C; (**d**) T = 10 °C; (**e**) T = 20 °C; (**f**) T = 40 °C.

The variation of damper force and deformation with service temperature is displayed in Figure 11. The peak forces of damper No. 40 are 163.71, 193.82, 224.87, and 284.08 kN, respectively, at 0, 10, 20, and 40 degrees Celsius. With the increase in service temperature, the peak damper force gradually increases, while the damper deformation decreases to some extent. Similar observations can be made for the other SMA dampers. The effects of service temperature on damper performance can also be found from the variation of damper hysteresis loops. The increasing service temperature may induce the increment in damper force and reduction in damper deformation. The enclosed areas of damper hysteresis loops reduce to some extent. Thus, a very large service temperature changes the damper states, and the damper may behave as a steel brace does and lose its energy-dissipating capacity. 

**Figure 12:**
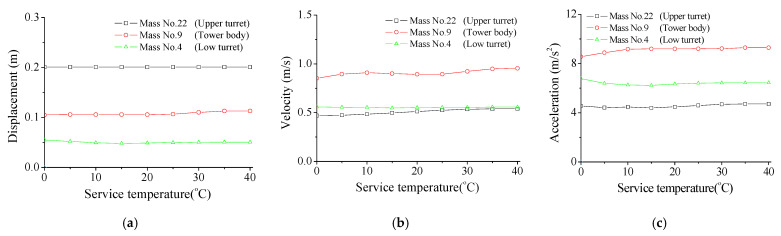
Effects of service temperature on maximum responses of the tower. (**a**) Displacement; (**b**) Velocity; (**c**) Acceleration.

The effects of service temperature on maximum responses of the TV transmission tower are studied, as shown in Figure 12. The responses of the tower top, mast, tower body, and two turrets are computed, respectively, under the common temperature range between 0 to 40 degrees Celsius. The displacement responses of the TV tower are almost stable with increasing service temperature, while the velocity and acceleration responses slightly increase. It is noted that a relatively large service temperature may affect the shape of damper hysteresis loops, as displayed in Figure 11, while the control performance is stable. This is because the peak forces of SMA dampers increase automatically with increasing temperature. 

### 6.4. Effects of Earthquake Intensity

**Figure 13:**
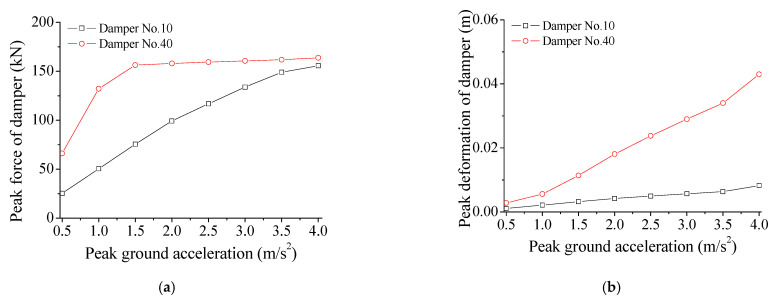
Variation of peak control force of SMA dampers with PGA. (**a**) Peak force; (**b**) Peak deformation.

The effects of PGA on control performance are investigated in this section. Figure 13 displays the variation of peak control force of SMA dampers with PGA. The deformation of SMA dampers almost increases linearly with the increasing PGA under constant damper stiffness. The peak control force of the SMA damper gradually increases with the increasing earthquake intensity. This means that the performance of SMA dampers presents certain robustness under external excitations. 

**Figure 14:**
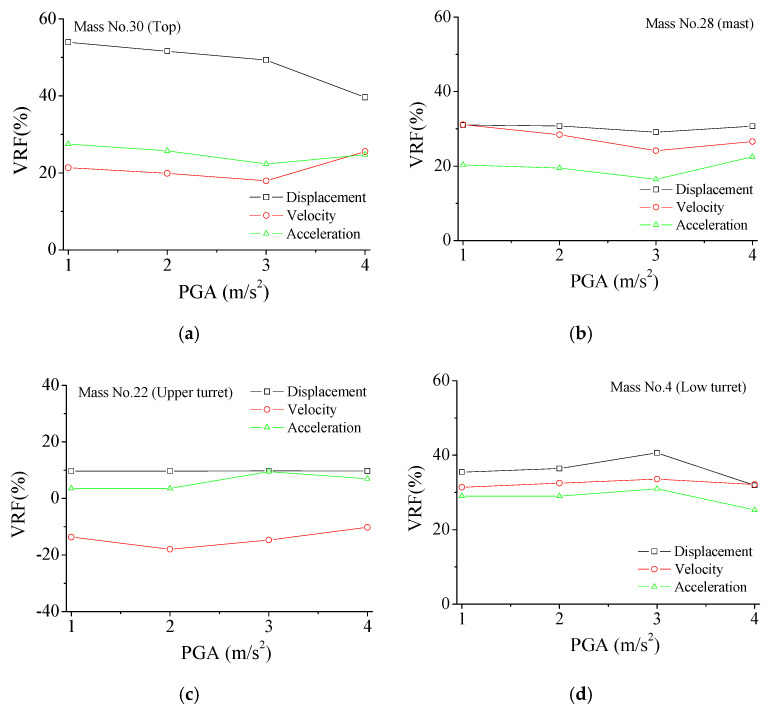
Variation of VRFs with seismic PGA. (**a**) VRF No. 30; (**b**) VRF No. 28; (**c**) VRF No. 22; (**d**) VRF No. 4.

As displayed in Figure 14, the displacement VRF of the tower top gradually increases, with the increment in PGA, while the velocity and acceleration VRFs almost keep constant. All three types of VRFs of the mast are stable with increasing earthquake intensity. Similar observations can be made for the upper turret and low turret. Thus, it is noted that the overall VRFs of the TV transmission tower are stable when subjected to different seismic excitations. The control performance by SMA dampers is robust and versatile for the earthquake-disturbed tower. 

### 6.5. Efficacy Subjected Other Earthquakes

The seismic responses under the other three earthquakes are also computed and compared with the same PGA of 4.0 m/s^2^. The response VRFs of the example tower under different earthquakes are listed in Table 4. Three far-field and two near-field historical records are selected to evaluate the performance of the proposed control method based on SMA dampers [32,33,34,35]. The SMA dampers can reduce structural seismic responses while the control efficacy, under different seismic inputs, is different. In addition, the overall VRFs of the tower top and mast are much better than those of the two turrets. Thus, the whiplash effects of the example tower can be substantially reduced. In reality, the different frequency components of the example earthquakes in the frequency domain induce the difference in structural dynamic responses. Therefore, control performances under different earthquakes cannot maintain optimality at all times.

### 6.6. Comparison between SMA Damper and Friction Damper

The dynamic responses of the tower with the commonly-used friction damper are computed and compared with those by SMA dampers. Friction dampers have been used to improve the seismic resistant performance of various structures during the past three decades. The Coulomb law is commonly adopted in the mathematical model of friction dampers. It presumes that the frictional force is independent of the velocity and the coefficient *μ* is the proportional constant considered in the mathematical relation between the frictional force and the clamping force *N*. The frictional force *f_d_* equals the clamping force *N* multiplies the coefficient of friction *μ*. The clamping force of a friction damper is set in advance, and its frictional force is constant during the slip process. If the force of the friction damper *f_d_* is no larger than the design frictional force, then the damper is sticking and the damper behaves as if it is a common brace. If the damper force *f_d_* is larger than the design frictional force, the damper begins to slip and dissipate vibrant energy. During the structural vibration under an earthquake, if the relative displacement increment of the two ends of the friction element is large than zero, the control force is tensile. Otherwise, the control force is compressive. As displayed in Figure 10b and Figure 11a, the peak force of an SMA damper is 150 kN. Thus, the peak control forces of friction dampers are set as the same for performance comparison. The number and location of friction dampers are also the same as those of SMA dampers. 

**Figure 15:**
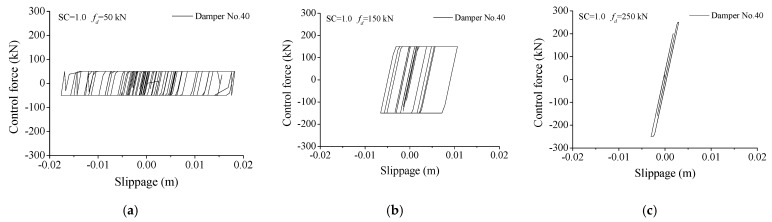
Hysteresis loops of friction dampers under different damper forces. (**a**) fd = 50 kN; (**b**) fd = 150 kN; (**c**) fd = 250 kN.

The hysteresis loops of friction dampers, under different damper forces, are displayed in Figure 15. For a friction damper, its damper force cannot be changed during the structural vibration. If the damper slipping force is too large (250 kN), the friction damper is hard to slip and behaves similarly to a pure brace. If the damper slipping force is too small (50 kN), the damper is very easy to slip while the enclosed area is small, which means a limited energy-dissipating capacity of the friction damper. Thus, the optimal slipping force of a friction damper depends on the intensity of the external excitations.

**Figure 16:**
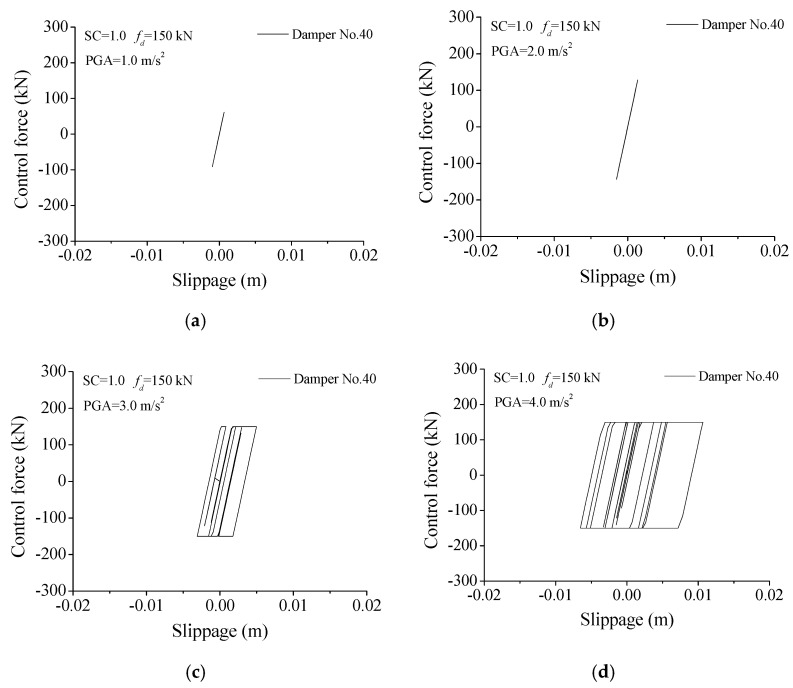
Hysteresis loops of friction dampers under different earthquake intensities. (**a**) PGA = 1.0 m/s^2^; (**b**) PGA = 2.0 m/s^2^; (**c**) PGA = 3.0 m/s^2^; (**d**) PGA = 4.0 m/s^2^.

The hysteresis loops of friction dampers, under different earthquake intensities, are displayed in Figure 16. For a small seismic intensity (PGA = 1.0 or 2.0 m/s^2^), the damper force of 150 kN is large enough, the damper is hard to slip, and it behaves as a brace. For an appropriate seismic intensity (PGA = 4.0 m/s^2^), the hysteresis loop is plump, and the control performance is satisfactory. For a larger seismic intensity (PGA = 5.0 m/s^2^), the damper can slip more easily, the enclosed area of the hysteresis loop is limited, and the energy-dissipating capacity and control performance is limited. The reason is that the slipping force of a friction damper is set in advance and cannot change with the varying excitation intensity. Therefore, the control robustness of friction dampers is unsatisfactory. 

**Figure 17:**
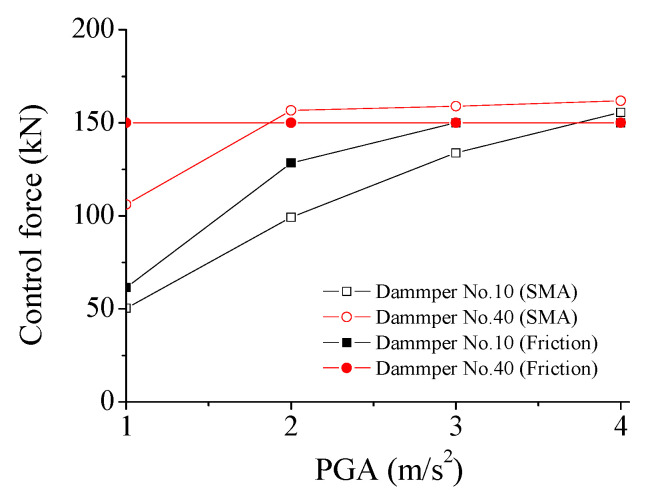
Variation of control force with seismic intensity.

The performance of SMA dampers is quite different in comparison with that of friction dampers. The variation of control force with seismic intensity is investigated and shown in Figure 17. It is seen that the control force of the SMA dampers can be modulated automatically with the earthquake-induced structural and damper responses. The control forces of SMA dampers quickly increase with the increasing seismic intensity. Thus, the SMA damper can move more easily under any seismic intensity. In addition, the shapes of hysteresis loops of SMA are similar, even if the seismic intensity increases. However, the performance of friction dampers is quite different in comparison with that of SMA dampers, as displayed in Figure 16 and Figure 17. The control forces of the friction dampers are set at 150 kN in advance and cannot be changed during the whole vibration. For a small seismic intensity (PGA = 1.0 or 2.0 m/s^2^), the friction dampers cannot slip, and they behave as a brace. Thus, the damper force is smaller than 150 kN. The energy-dissipating capacity of SMA dampers is stable and superior to that of friction dampers.

The structural VRFs, under different seismic intensities, are computed and compared, as displayed in Table 5. It is observed that the VRFs of SMA dampers are almost stable for different seismic intensities. The SMA damper-based control approach is robust and versatile for the TV transmission tower. However, the control efficacy of friction dampers with constant slipping force (150 kN) may dramatically reduce with the varying seismic intensity. If the damper force is a certain value and cannot be automatically changed, a friction damper is easy in the sticking stage and behaves much as a common brace does under a small seismic intensity. Thus, the friction damper is unable to fully slip, which causes unsatisfactory control performance. However, an SMA damper with proper parameters can automatically adjust its control force in line with the change of external loading and thereby ensure robust control performance. 

## 7. Energy Responses of the Tower

### 7.1. Comparison of Energy Responses of the Tower

**Figure 18:**
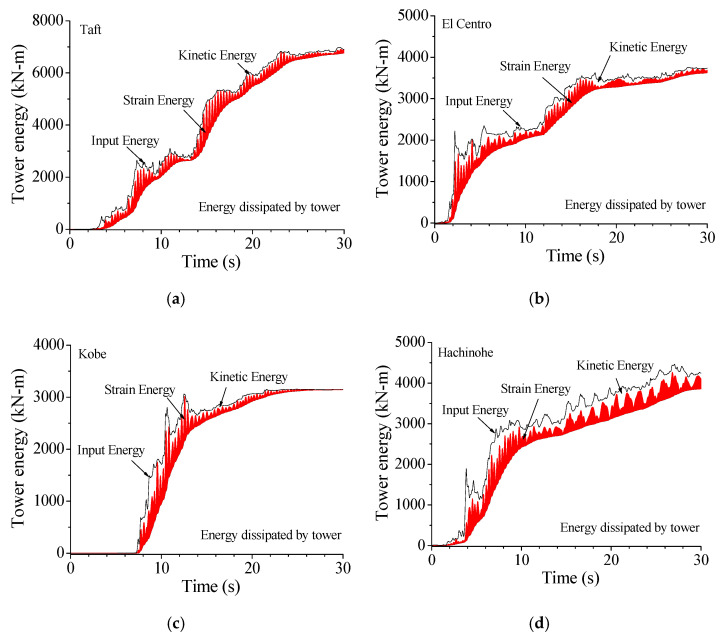
Energy responses of the tower without friction dampers. (**a**) Taft; (**b**) El Centro; (**c**) Kobe; (**d**) Hachinohe.

The vibrant intensity of the TV transmission tower can also be depicted from the viewpoint of energy. The energy properties of the example tower, subjected to various earthquakes, are displayed in Figure 18. For the uncontrolled tower under the Taft earthquake, the total inputted energy from the seismic excitations is absorbed by structural damping. Large kinetic and strain energy is seen because of the excessive vibration of the tower. Similar observations can be made from the other three ground motions. The magnitude of total inputted energy to the tower, under various earthquakes, is quite different even though their PGAs are the same. Thus, the frequency components of seismic excitations have substantial influences on structural dynamic responses.

**Figure 19:**
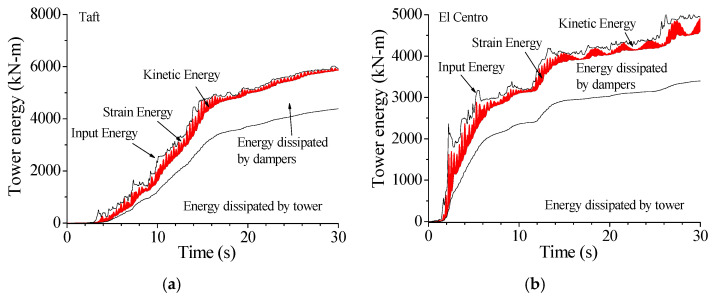

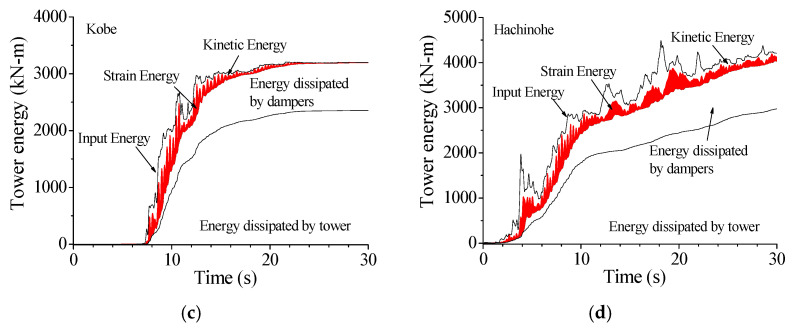
Energy responses of the tower with SMA dampers. (**a**) Taft; (**b**) El Centro; (**c**) Kobe; (**d**) Hachinohe.

Figure 19 displays the energy responses of the tower with SMA dampers in comparison with those without control. The application of SMA dampers can substantially reduce the vibrant energy of the TV transmission tower to a great extent. To compare the energy curves in Figure 18 and Figure 19, one can find that the inputted energy of the controlled tower is smaller in comparison with the counterpart of the controlled tower. Owing to the aid of SMA dampers, the energy absorbed by structural damping is much less than that of the uncontrolled tower. The energy responses under Northridge earthquake have the similar properties. 

### 7.2. Variation of Energy Response with Damper Stiffness

**Figure 20:**
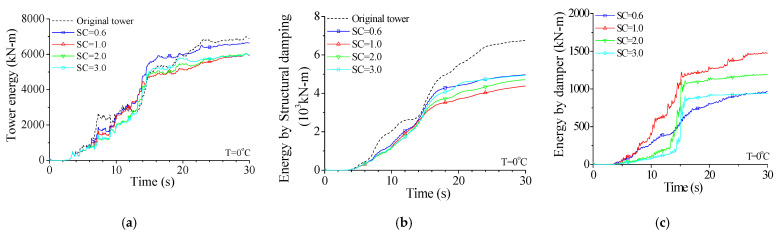
Variation of energy responses with damper stiffness. (**a**) Tower energy; (**b**) Energy by structural damping; (**c**) Energy by damper.

Curves in Figure 20 indicate that the total inputted energy from earthquakes *E_E_* varies with the damper stiffness. For a small damper stiffness (SC = 0.6), the energy-dissipating capacity of the SMA dampers is poor, and the tower energy *E_E_* is even slightly larger than that of the uncontrolled tower. The tower energy *E_E_* gradually reduces with the increasing SC until approximately 1.0. A further increase in damper stiffness (SC = 2.0 or 3.0) may induce a large value of *E_E_*. However, the energy dissipated by SMA dampers *E_P_* is quite different. As displayed in Figure 20, *E_P_* gradually increases with the increasing damper stiffness until SC reaches about 1.0. If stiffness coefficient SC reaches above 2.0, *E_P_* reduces to some extent. The energy dissipated by SMA dampers *E_P_* is limited if the damper stiffness is relatively small (SC = 0.6) or large (SC = 2.0 or 3.0). Besides, similar observations can be made from the energy curves of structural damping *E_D_*. With the increase in damper stiffness, the energy *E_D_* gradually reduces because more structural energy *E_E_* is dissipated by SMA dampers. Thus, optimal damper stiffness can also be determined based on structural energy curves.

## 8. Concluding Remarks

The validity of the proposed control approach for TV transmission towers by SMA dampers is examined in detail. The PSD properties and energy responses of the tower are compared with those of the uncontrolled tower. The results indicate that SMA dampers, with optimal parameters, can be used in the control of TV transmission towers disturbed by earthquakes. 

Dynamic responses of the TV tower are remarkably suppressed by SMA dampers particularly for the whiplash effects of the top mast. The overall control efficacy of scheme 1 is better than that of the other two schemes, which means that the optimal positions for SMA dampers are both two turrets. The hysteresis loops of SMA dampers are quite different because the damper stiffness can directly affect the damper’s working status. The optimal damper stiffness can also be determined based on the configuration of hysteresis loops. The vibrant energy of the controlled tower is substantially reduced, and the inputted energy of the controlled tower is also mitigated to a great extent. The application of SMA dampers can remarkably improve the energy-dissipating mechanism of the TV transmission tower. Energy evaluation is an effective approach to determine the optimal damper parameters in vibration control.

It is not beneficial to set a very large SC value for SMA dampers to avoid unnecessary cost waste. Thus, an optimal SC value for all the responses does not exist, and optimal SC values for three kinds of peak responses at different places are different to some extent. Thus, in the real application of SMA dampers, an optimal damper stiffness should be determined based on the structural model of TV transmission tower through detailed parametric study, which may be time-consuming and complicated. Similarly, an optimal service temperature is required based on the structural model of the TV transmission tower through a detailed and complicated parametric study.

## Figures and Tables

**Table 1:** Comparison of dynamic properties of the 3D and 2D models.

Freq.no	3D Finite Element Model	2D Dynamic Model	Error
1	0.129	0.128	0.621%
2	0.416	0.386	7.211%
3	0.729	0.781	−7.133%
4	1.171	1.163	0.683%
5	1.514	1.456	3.831%
6	2.221	2.142	3.556%
7	2.578	2.569	0.349%
8	2.632	2.783	−5.737%

**Table 2:** Physical parameters of SMA dampers.

Parameter	Value	Parameter	Value	Parameter	Value
*M*_*f*_	−46 °C	*C*_*M*_	10 MPa/°C	$\sigma_{s}^{cr}$	120 MPa
*A*_*s*_	−18.5 °C	*C*_*A*_	15.8 MPa/°C	$\sigma_{f}^{cr}$	190 MPa
*M*_*s*_	−37.4 °C	*D*_*A*_	75,000 MPa	*ε*_*L*_	0.079
*A*_*f*_	−6 °C	*D*_*M*_	29,300 MPa		

**Table 3:** Damper installation schemes.

Scheme 1	Scheme 2	Scheme 3
30 dampers around the low turret (Mass No. 4) 30 dampers around the upper turret (Mass No. 24)	60 dampers around the low turret (Mass No. 4)	60 dampers around the upper turret (Mass No. 24)

**Table 4:** VRFs of the TV transmission tower under different earthquakes (%).

Location		Taft	El Centro	Kobe	Hachinohe	Northridge
Mass No. 30 (Tower top)	Displacement	39.64	31.95	22.38	23.24	29.03
	Velocity	25.57	13.87	18.70	33.89	5.59
	Acceleration	24.75	6.43	21.09	13.89	11.36
Mass No. 28 (Mast)	Displacement	30.70	19.46	23.64	22.56	10.70
	Velocity	26.59	−5.21	20.69	19.60	11.42
	Acceleration	22.48	−9.28	17.52	18.51	15.04
Mass No. 22 (Upper turret)	Displacement	9.72	9.24	8.31	8.91	10.77
	Velocity	−10.19	11.88	23.94	−1.05	−0.78
	Acceleration	6.95	8.86	10.48	−4.56	5.55
Mass No. 4 (Low turret)	Displacement	31.88	−2.81	35.34	2.69	4.04
	Velocity	32.14	−10.05	23.30	1.08	0.42
	Acceleration	25.30	−5.56	15.60	8.74	−1.64

**Table 5:** Comparison of VRFs with different control approaches under different earthquake intensities.

Location		SMA Damper PGA = 4.0	Friction Damper PGA = 4.0	SMA Damper PGA = 1.0	Friction Damper PGA = 1.0
Mass No. 30 (Tower top)	Displacement	39.64	39.87	53.92	40.69
	Velocity	25.57	38.84	21.37	33.72
	Acceleration	24.75	52.63	27.49	37.30
Mass No. 28 (Mast)	Displacement	30.70	47.07	30.99	8.61
	Velocity	26.59	49.51	31.12	31.33
	Acceleration	22.48	44.75	20.33	23.28
Mass No. 22 (Upper turret)	Displacement	9.72	8.61	9.68	8.57
	Velocity	−10.19	−3.93	−13.67	−5.77
	Acceleration	6.95	9.11	3.529	9.08
Mass No. 4 (Low turret)	Displacement	31.88	39.43	35.471	33.37
	Velocity	32.14	30.80	31.37	36.74
	Acceleration	25.30	29.61	29.02	23.16

## Data Availability

Data sharing is not applicable to this article.
